# Surface Superconductivity Changes of Niobium Sheets by Femtosecond Laser-Induced Periodic Nanostructures

**DOI:** 10.3390/nano10122525

**Published:** 2020-12-16

**Authors:** Álvaro Cubero, Elena Martínez, Luis A. Angurel, Germán F. de la Fuente, Rafael Navarro, Herbert Legall, Jörg Krüger, Jörn Bonse

**Affiliations:** 1 Instituto de Nanociencia y Materiales de Aragón (INMA), CSIC-Universidad de Zaragoza, 50009 Zaragoza, Spain; acubero@unizar.es (Á.C.); angurel@unizar.es (L.A.A.); german.delafuente.leis@csic.es (G.F.d.l.F.); 2 Bundesanstalt für Materialforschung und -prüfung (BAM), Unter den Eichen 87, 12205 Berlin, Germany; herbert.legall@bam.de (H.L.); joerg.krueger@bam.de (J.K.); joern.bonse@bam.de (J.B.)

**Keywords:** niobium, surface superconductivity, laser-induced periodic surface structures (LIPSS), femtosecond n-IR laser

## Abstract

Irradiation with ultra-short (femtosecond) laser beams enables the generation of sub-wavelength laser-induced periodic surface structures (LIPSS) over large areas with controlled spatial periodicity, orientation, and depths affecting only a material layer on the sub-micrometer scale. This study reports on how fs-laser irradiation of commercially available Nb foil samples affects their superconducting behavior. DC magnetization and AC susceptibility measurements at cryogenic temperatures and with magnetic fields of different amplitude and orientation are thus analyzed and reported. This study pays special attention to the surface superconducting layer that persists above the upper critical magnetic field strength *H*_c2_, and disappears at a higher nucleation field strength *H*_c3_. Characteristic changes were distinguished between the surface properties of the laser-irradiated samples, as compared to the corresponding reference samples (non-irradiated). Clear correlations have been observed between the surface nanostructures and the nucleation field *H*_c3_, which depends on the relative orientation of the magnetic field and the surface patterns developed by the laser irradiation.

## 1. Introduction

It is well established for type II superconductors that both a lack of material’s extended lattice periodicity (grain boundaries, stacking faults, etc.) and local crystallographic defects (vacancies, substitutions) interact with magnetic vortices and act as effective pinning centers of the magnetic flux lines. The vortex pinning forces generated by these structural imperfections cause flux density gradients that contribute to the irreversible behavior of the magnetization [1] and to low frequency AC losses. Vortex pinning is a phenomenon of great relevance for practical conductors since it allows the superconductor to carry resistance-less current.

Type II superconductors with negligible bulk pinning may also present hysteresis effects, due to Bean–Livingston surface barriers [2] or due to geometrical edge barriers (specimen-shape dependent) [3]. Both types have been observed in low- and high-temperature superconductors (LTS and HTS, respectively) [2,3,4,5,6,7,8]. The former type of barriers is generally observed in clean single crystals, whereas the latter is more pronounced for thin films of constant thickness in perpendicular magnetic fields. In both cases, the magnetic irreversibility is caused by the asymmetry between the magnetic flux entry and exit. In type II superconductors, vortex cores overlap at the upper critical field strength, *H*_c2_, and superconductivity becomes extinguished from the bulk. However, it is worth noting that a surface superconducting layer can persist above *H*_c2_, up to the surface critical field strength, *H*_c3_. For flat surfaces, Saint James and De Gennes [9] predicted a superconducting layer of a thickness approximately equal to the superconducting coherence length, up to a field *H*_c3_ ≈ 1.69 × *H*_c2_ when the field is applied parallel to the superconductor’s surface. These surface current vortices can be pinned, resulting in a surface critical current (*i*_c_), which depends on the surface characteristics, such as roughness and morphology. These can be changed, for example, by different polishing procedures [10,11,12], or by low-energy Ar^+^ ion irradiation of the surface [13].

Within the class of type II LTS, pure niobium has been widely studied in the literature by its intrinsic properties and by its application in superconducting radio frequency (SRF) cavities, where surface control has the greatest relevance. In SRF applications, very clean surfaces are required to achieve a high quality factor, *Q*_0_, which is inversely proportional to the surface resistance [11]. Surface treatment procedures including thermal etching, electropolishing and buffered chemical polishing are usually used to achieve the former requirement [10,12]. Grassallino et al. [14] also found that the annealing in a partial pressure of nitrogen or argon gas, followed by the electropolishing of the niobium cavity, yields very low values of the microwave surface resistance and, therefore, more efficient accelerating structures.

Nowadays, focused laser pulsed sources allow surface control on the near micrometer and submicrometer scales, enabling surface specialized functionalities in a wide variety of materials and with increasing processing speeds. Different processing techniques, such as laser direct writing, laser interference patterning or laser-induced self-organization, enable the control and modification of the laser-processed surface morphologies [15,16,17,18,19,20,21]. Particularly here, a laser-induced self-ordering process [22] has been chosen that enables the generation of laser-induced periodic surface structures (LIPSS) in a robust single-step approach. In general, such surface structures may exhibit the shape of grating-like ripples, grooves, spikes, pillars, cones, etc., featuring spatial periods ranging from a few tens of micrometers to a few tens of nanometers far beyond the wavelength diffraction limit [17,18,21,22]. Furthermore, femtosecond (fs) laser pulses facilitate the attainment of LIPSS with minimum thermal heating effects on the irradiated target.

Ultra-short laser processing, thus, opens the possibility of changing the surface morphology through periodic surface structures on large areas and in continuous fabrication processes featuring currently maximal areal processing rates at the m^2^/min level for both the laser interference patterning and the self-organization approaches [23]. Moreover, the spatial period of the LIPSS can be controlled via the laser irradiation wavelength, the laser pulse fluence or by the effective number of incident pulses [21]. Particularly, close to the ablation threshold of fs-laser irradiated metals, a significant variation of the ripple period can be realized [24,25,26]. Hence, the localized generation and control of surface defects, such as ripples, is a promising approach to affect the surface superconducting properties.

In this work, two different near-infrared (n-IR) fs-lasers, which differ in wavelength and pulse duration, have been used for laser structuring of Nb foil samples. Ultra-short laser irradiation produces distinctive quasi-periodic nanostructures that vary with the laser pulse fluence or with the effective number of incident pulses. Here, these values have been chosen to produce elongated quasi-parallel morphologies of ripples that are not isotropic. The microstructural changes following irradiation have been analyzed and compared with non-irradiated samples. The effects of applying different atmospheres (argon, nitrogen and air) during the laser treatment process have also been analyzed. In a previous work, it was reported that the laser-generated structures on Nb foils produce some irreversible changes in the magnetic behavior of this superconductor [18]. Nevertheless, discriminating between bulk and surface effects frequently presents important challenges. The aim of the present work is to study the effect of femtosecond n-IR laser structuring of Nb foil samples on their surface superconductivity characteristics. These properties were characterized using DC magnetization and AC susceptibility measurements with the magnetic field applied parallel to the Nb foil surface in order to maximize *H*_c3_ values and to minimize the geometric specimen-shape factor. The effect of the LIPSS anisotropy on the surface superconductivity behavior has been studied and is reported here.

## 2. Materials and Methods

Commercial Nb foil samples (rolled, 25 μm thickness, 99.8% purity and typical roughness of 0.30–0.35 μm) (Sigma-Aldrich, Darmstadt, Germany) have been irradiated with two different n-IR femtosecond lasers located in different laboratories, i.e., at the Institute of Nanoscience and Materials of Aragón (INMA) in Zaragoza (Spain), and at the German Federal Institute for Materials Research and Testing (BAM) in Berlin: (i) L1 at INMA: n-IR Yb:YAG laser (Light Conversion, Vilnius, Lithuania), (Carbide CB3-40W), center wavelength λ = 1030 nm, pulse duration *τ*_p_ = 280 fs. The focusing of the laser beam was realized by means of a cylindrical lens system (focal length of 150 mm) leading to an elliptical beam with 1/e^2^ diameters of 2*a*_b_ = 1500 μm and 2*b*_b_ = 26 μm. (ii) L2 at BAM: n-IR Ti:Sapphire laser (Femtolasers, Vienna, Austria) (Compact Pro), center wavelength λ = 790 nm, pulse duration *τ*_p_ = 30 fs. For properly handling such extremely short femtosecond laser pulses, the focusing of the laser beam was realized by a spherical dielectric mirror (Layertec GmbH, Mellingen, Germany) (focal length of 500 mm) resulting in a circular beam of 1/e^2^ diameter *D*_b_ = 2 *r*_b_ = 130 μm. For both laser systems, the experiments were performed with a pulse repetition frequency *f*_rep_ = 1 kHz and the focused 1/e^2^ beam diameters were determined in the sample processing plane using the *D*^2^-method proposed by Liu [27,28]. At the given conditions, the areal processing rates were approximately 1.0 mm^2^/s for L1 and 0.12 mm^2^/s for L2 here. For irradiation with L1, the sample was placed inside a chamber that allows laser processing in different gaseous atmospheres, such as air, Ar or N_2_; and the line-wise laser scanning was performed in the direction perpendicular to the major axis of the elliptical beam. In all experiments, the laser scanning direction coincides with that of the linear laser beam polarization.

Table 1 collects the laser processing conditions of the irradiated Nb samples. With laser L1, two samples were processed with the same conditions but changing the atmosphere (Ar and N_2_). In both cases, the laser polarization was parallel to the rolling direction. The two samples that were processed with laser L2 were treated in air. The difference between samples “FS_air1” and “FS_air2” is that they were irradiated with orthogonal orientation with respect to the Nb foil rolling orientation. Sample FS_air2 sample was oriented in order to have laser beam polarization perpendicular to the rolling direction. A reference non-irradiated sample, named “REF”, was also studied for comparison.

The main parameters of laser processing were described in detail in [18] but are summarized here as follows. *P* is the nominal average laser power and *f*_rep_ the pulse repetition frequency. Each laser pulse is characterized by the peak fluence (*F*_p_) and the peak irradiance (*I*_p_). For a given value of *f*_rep_, the laser beam scanning velocity (*v*_L_) controls the distance between two laser pulses in a line, *d*_p_ = *v*_L_/*f*_rep_. Taking into account the Gaussian beam profile in the processing plane, a uniform fluence distribution is obtained in the full 2D scanned area if line-to-line overlap is above a critical threshold value (*d*_s_/*r*_b_ for L2 or *d*_s_/*a*_b_ for L1 is below 0.9, where *d*_s_ is the distance between adjacent lines). This is fulfilled for the treatments performed with laser L2, where *d*_s_ is 20 μm while the characteristic beam radius is *r*_b_ = 65 μm. In this situation, the total accumulated fluence can be calculated as

(1) $$F_{2D}=\frac{\pi \;r_{b}^{2}}{d_{p}\;d_{s}}\;F_{p}$$

Laser parameters were selected in order to have similar *F*_2D_ values with both lasers. A constant value of *F*_2D_ = 16.6 J/cm^2^ was obtained for samples treated with laser L2. In the case of laser L1, the distance between two lines (*d*_s_ = 1 mm) is larger than the characteristic line size (*a*_b_ = 0.75 mm) leading to a non-uniform fluence distribution on the surface, with an average value of 17.1 J/cm^2^.

The optical penetration depth 1/*α* = λ/(4π*k*), with *α* being the linear absorption coefficient and *k* the imaginary part of the complex valued refractive index, accounts to ~16 nm at both laser irradiation wavelengths.

The surface microstructural characterization was performed in a MERLIN field-emission scanning electron microscope (FE-SEM) (Carl Zeiss, Jena, Germany) equipped with an energy dispersive X-ray spectroscopy (EDX) system (Oxford Instruments, Abingdon, UK) operated at 5 kV. Surface topographic cross-sections were analyzed by Scanning Transmission Electron Microscopy (STEM) using a Tecnai F30 microscope (FEI Company, Hillsboro, OR, USA), also equipped with a high-angle annular dark field (HAADF) detector. Sample preparation was performed with a Focused Ion Beam (FIB) in a Dual Beam Helios 650 (FEI Company, Hillsboro, OR, USA) apparatus, using 30 kV Ga^+^ ions for the initial steps and 5 kV for final thinning. Prior to the preparation of a FIB lamella, a protective Pt cap layer was deposited at the region of interest. X-ray photoelectron spectroscopy (XPS) was applied to characterize the changes in the chemical state of the surface using an AXIS Supra spectrometer (Kratos, Manchester, UK). The photoemission was excited with monochromatic Al K_α_ X-ray radiation at 1486 eV over a spot size of 700 × 300 µm^2^, resulting in an XPS information depth between 5 and 10 nm. The carbon C1s peak at 284.8 eV served as the reference signal for energy calibration.

**Figure 1:**
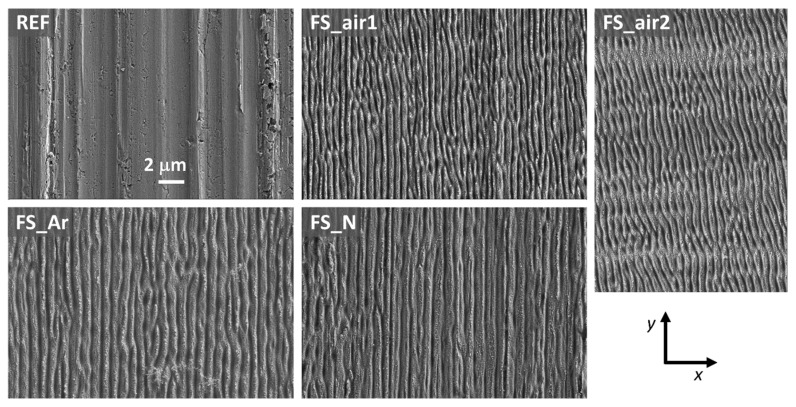
FE-SEM micrographs (secondary electrons) of the surfaces of all analyzed representative samples (same magnification for all images). The rolling direction is parallel to the *y*-axis in all samples, except for FS_air2, where it is parallel to *x*. The linear laser beam polarization is parallel to *x* in all cases.

Measurements of DC magnetization (*M*) and complex AC susceptibility, *χ*_ac_ (with in-phase, *χ*′, and out-of-phase, *χ*″, components) were carried out in a SQUID-based MPMS-5T system (Quantum Design, San Diego, CA, USA). The Reciprocating Sample Option (RSO) of the system was used for DC measurements. For *χ*_ac_ measurements, the AC drive magnetic field (sine wave of amplitude μ_0_*h*_0_ and frequency *f*) is superimposed on the constant DC magnetic field, μ_0_*H*. In this work, AC and DC components of the applied magnetic field have the same direction. Values of *f* = 10 Hz and μ_0_*h*_0_ = 10 and 100 μT have been used. The magnetic field was applied parallel to *x*- and *y*- axes of the foil’s surface plane (see Figure 1), in order to analyze if laser polarization and rolling orientations have any effect on their magnetic and superconducting behavior. For magnetic measurements, the size of the measured sample area is in the range of (3–4) mm × (3–4) mm and both surfaces of the samples were irradiated. The critical temperature (*T*_c_) was determined as the onset of diamagnetism from *χ*′(*T*), being *T* the temperature, obtaining *T*_c_ values ranging between 9.30 and 9.35 K in all samples. The heat capacity was measured in a PPMS-9T (Quantum Design, San Diego, CA, USA) apparatus.

## 3. Results and Discussion

### 3.1. Microstructural Characterization

Figure 1 shows SEM micrographs from the surface of each of the five analyzed samples. The reference sample clearly shows the microstructural characteristics arising from rolling, with defects parallel to the rolling direction (*y*-axis in the figure). These are also visible, although with less clarity, after laser irradiation. This rolling direction coincides with the *y*-axis in all the samples, except in the case of sample FS_air2, which was rotated by 90°. Surface irradiation of the samples with both fs-lasers produces, in all cases, elongated quasi-periodic surface structures aligned perpendicular to the laser polarization (direction *x* in the figure). This type of near-wavelength sized ripples is known as low spatial frequency LIPSS (LSFL) [17,22]. These structures are caused by the excitation of surface plasmon polaritons (SPPs) at the rough metallic surface and their interference with the incident laser radiation. The intra-pulse interference modulates the spatial pattern of optical energy absorbed by the electronic system of the solid and leads—after electron-phonon energy relaxation—to spatially modulated ablation [22]. This formation mechanism is supported by the values of the dielectric permittivity of the material Nb at the laser irradiation wavelengths that account to *ε* = −10.1 + i × 15.6 at 790 nm and *ε* = −24.4 + i × 16.8 at 1030 nm, respectively [29]. Hence, at both irradiation wavelengths, the condition R(ε) < −1 is fulfilled here—a prerequisite for the excitation of SPPs [30,31]. The relevance of specific hydrodynamically driven supra-wavelength sized ripples that were observed upon irradiation of thin metal films on dielectric substrates with high aspect ratio elliptical ns-laser beams parallel to the direction of scanning [32] can be ruled out here, since, in our case, the ripples (LSFL) are formed perpendicular to the direction of beam scanning—always perpendicular to the laser beam polarization and they exhibit near wavelength sized periods (see Figure 1).

The EDX analyses of the sample surfaces revealed an increase in the % of O elemental composition in the samples processed in air (6.2 wt%, 27.7 at%) compared to the reference sample (4.1 wt%, 19.9 at%), and a reduction for samples processed in N_2_ or Ar (3.4 wt%, 17.0 at%). The standard deviations of the wt% values range between 0.2 and 0.4. A small amount of nitrogen (0.6 wt%, 3.0 at%, 0.1 wt% sigma) was detected in sample FS_N. Note that the absolute percentage values must be taken with care here due to the large EDX information depth and due to surface corrugations being present in the laser processed areas.

**Figure 2:**
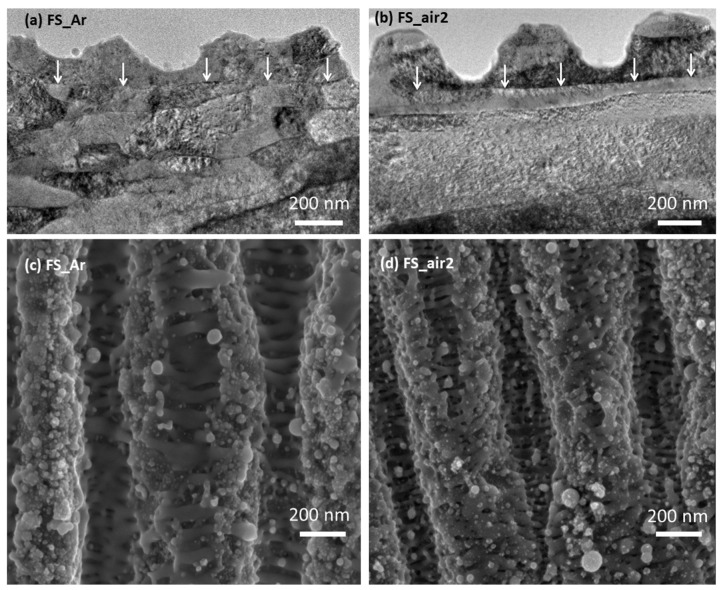
(**a**,**b**) STEM micrographs of the cross-sectional images near the surface. (**c**,**d**) FE-SEM top-view images (in-lens detector) of the surface of the same samples. (Left column) FS_Ar, (Right column) FS_air2. The white arrows in (**a**,**b**) point at the boundary between laser-affected and non-affected regions.

Quasi-periodic submicrometer-structures induced by both lasers can be better analyzed using higher magnification, as it is demonstrated in Figure 2 for samples FS_Ar and FS_air2. The upper row images in the figure correspond to cross-sectional views obtained by STEM for a lamella extracted from the samples (cut parallel to *x*-axis). The lower row images correspond to surface views by FE-SEM using an in-lens secondary detector. LSFL were formed in both samples and have similar modulation depths (peak-to-valley distances of about 200 nm). These structures are more homogeneous for sample FS_air2, as it is clearly seen in the cross-sectional view. The spatial period of the LSFL is 775 nm for samples irradiated with laser L1 and 570 nm for those irradiated with laser L2. These are mean values that have been calculated by analyzing statistically representative areas observed on samples by FE-SEM, and have standard deviations of 68 and 35 nm, respectively. The differences in the observed periods are assigned to the different laser wavelengths emitted by L1 and L2. It must be pointed out that the geometrical characteristics of the LSFL are in good agreement with the atomic force microscopy measurements published for fs-laser irradiated niobium in [18,33]. High spatial frequency LIPSS (HSFL) with periods of 50 to 80 nm, which form between LSFL structures, being perpendicular to them, are also observed in both samples (see Figure 2c,d), in line with the observations reported in [34].

It is important to note here that, due to the rolling process involved in the manufacturing of the Nb foils, the grains are elongated in the bulk of the material, with the longest grain axis oriented parallel to the rolling direction. This is also parallel to the ripple orientation in sample FS_Ar, but perpendicular in sample FS_air2. This is the reason for the different grain shapes observed in Figure 2a,b, with a clear texture of grains oriented perpendicular to the nanostructure observed in sample FS_air2. Interestingly, a boundary can be observed in both samples that is separating the laser-affected region from the non-affected bulk material underneath (marked by white arrows in Figure 2a,b. Supposedly, the Nb surface was melted up to this depth during the fs-laser scan processing, resulting in a re-solidified layer of 40 to 300 nm here (depending on the position across the LIPSS). This thickness is larger than the optical penetration depth of the laser radiation in Nb, which can be explained by the multi-pulse laser treatment upon scan-processing.

**Figure 3:**
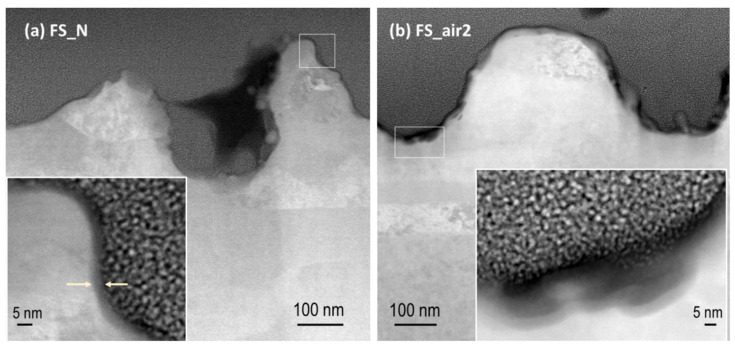
HAADF-STEM images of a cross-section of sample (**a**) FS_N and (**b**) FS_air2, near the surface. The insets show the areas highlighted by a white rectangle in the corresponding main images, with higher magnification.

Figure 3 shows HAADF-STEM images of the cross-section of samples FS_N and FS_air2. Corresponding STEM-EDX analyses confirm the presence of O and Nb elements in the darker areas observed near the sample surface (data not shown here). These zones are more abundant in the sample processed in air (Figure 3b) than in the samples processed in nitrogen (Figure 3a) or argon (not shown here). The oxide layer can be associated with the dark interfacial zones and exhibits a thickness of a few nanometers for the sample FS_N (≈5 nm in the zone shown in the inset of Figure 3a). For sample FS_air2, however, this layer exhibits an increased thickness (reaching values up to 20 nm) and is much less uniform. Note that the thickness of the oxide layer formed upon fs-laser processing in nitrogen here is very similar to that found for the natural Nb passivation layers that are characterized by a thickness of about 6–8 nm and Nb_2_O_5_ as the outermost layer [33].

**Figure 4:**
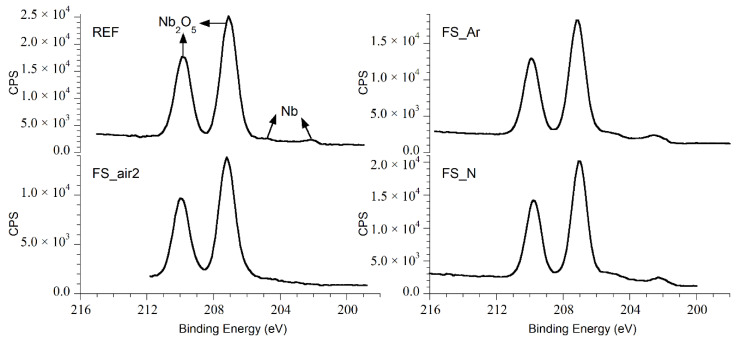
High resolution Nb 3d XPS spectra for the analyzed samples discussed in the text. CPS: counts per second.

**Figure 5:**
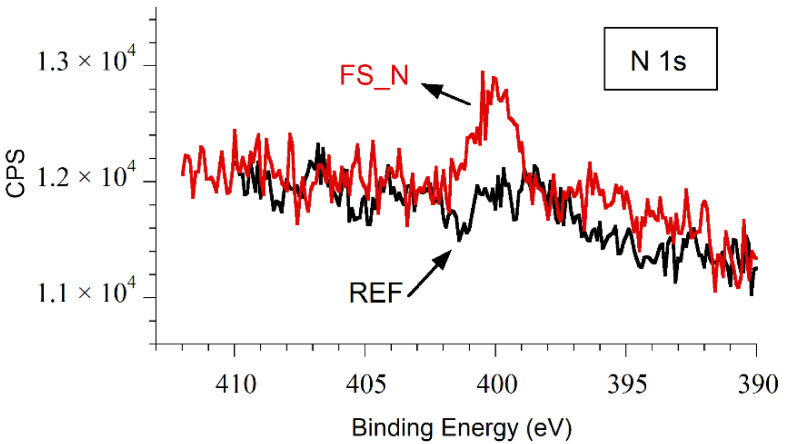
High resolution XPS spectra of the N 1s line of samples REF (non-irradiated, black line) and FS_N (laser irradiated in nitrogen atmosphere, red line). CPS: counts per second.

The high resolution XPS spectra of niobium Nb 3d, plotted in Figure 4, show very similar behavior for all samples, with the presence of the Nb 3d doublet at binding energies (BEs) of 209.80 and 207.05 eV, corresponding to Nb_2_O_5_, as the main chemical compound at the surface [35,36,37]. The peaks corresponding to metallic Nb, Nb^0^, are also observed in all samples except in the one processed in air, FS_air2. The latter indicates a thickness of the laser-induced oxide layer exceeding the XPS information depth here—fully in line with Figure 3b and with previous observations made for fs-irradiation of Ti in the air environment [38]. The sample irradiated in nitrogen atmosphere exhibits a very weak signal in the N 1s spectrum with a BE of approximately 400.0 eV (see Figure 5), thus discarding the presence of niobium nitrides, NbN_x_, which are expected to appear at lower binding energies (396.5 eV [39], 397.5 eV [40]). This is very similar to the results reported for nitrogen doped niobium [37], where the peak at 399.84 eV in the N 1s spectrum was attributed to the formation of CH_3_CN.

### 3.2. Irreversible Magnetization and Upper Critical Field, H_c2_

**Figure 6:**
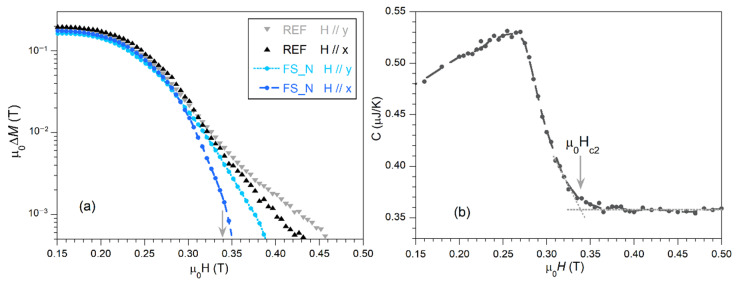
(**a**) Field dependence of the magnetic hysteresis loop width, μ_0_Δ*M*(*H*) at *T* = 5 K of the REF and FS_N samples with the external DC field applied in the *x*- and *y*-directions, both parallel to the surface as indicated in Figure 1; (**b**) heat capacity of the non-irradiated reference (REF) sample recorded at 5 K. The derived upper critical field μ_0_*H*_c2_ is marked by an arrow. Both arrows point to the same magnetic field, for better comparison.

Figure 6a shows the width of the magnetic hysteresis loop, Δ*M*, as a function of the magnetic field for the reference non-irradiated sample (REF) and the irradiated sample (FS_N), at the temperature *T* = 5 K, and with the magnetic field applied parallel to the *x*- and *y*-axes. Δ*M* was obtained for each *H* value as Δ*M* = *M*_↓_ − *M*_↑_, where *M*_↑_ and *M*_↓_ are the corresponding values measured for increasing and decreasing fields, respectively.

In these measurements, the sharp increase of the magnetic irreversibility, which is related to Δ*M*, is frequently associated to the onset of bulk superconductivity and, therefore, to the upper critical field μ_0_*H*_c2_ [10]. Nevertheless, this value is not always easy to derive from magnetization curves, due to the appearance of a tail at high magnetic fields in some conditions, as in this case [41]. With this aim, heat capacity, which is essentially a bulk property, could better allow the determination of μ_0_*H*_c2_, as marked by the arrow in Figure 6b [12,13]. It must be noted that the *C*(*H*) curve for the FS_N sample (not shown here for clarity reasons) is very similar to that of the reference sample, indicative of similar bulk properties (and μ_0_*H*_c2_) between both samples.

The results in Figure 6a thus clearly show irreversible magnetization values above μ_0_*H*_c2_, with differences between samples and orientations. The existence of non-zero Δ*M* values above μ_0_*H*_c2_ is indicative of the presence of surface critical currents, *i*_c_. As it is observed, fs-laser irradiation produces a significant decrease of Δ*M* above *H*_c2_, particularly when the magnetic field is applied in the direction perpendicular to the nanostructures (*x*-direction, as indicated in Figure 1). This effect is observed for all analyzed irradiated samples, independently of the processing atmosphere. It must be noted that the reference sample also exhibits some anisotropy in Δ*M*, with higher values above *H*_c2_ for the field applied parallel to the rolling direction (*y*-axis). This is indicative of an influence of the anisotropy of the microscopic grain structure induced by rolling, as visualized in Figure 2a,b and Figure 3, in agreement with previous studies [41].

Changes in the Δ*M* (or *i*_c_) at fields above *H*_c2_ for different surface treatments have been reported by several groups. For example, Scola et al. [13] observed an increase of *i*_c_ values after irradiating the Nb surface with low-energy Ar^+^ ions; Aburas et al. [12] observed changes by polishing the surface using different processes (sandpaper, diamond, colloidal silica and chemical polishing), with lower *i*_c_ values for smoother surfaces; Casalbuoni et al. [10] also reported differences in *i*_c_ values of Nb cylinders subjected to buffered chemical polishing or to electropolishing; and van Gurp [41] observed higher *i*_c_ values for cold rolled Nb foils compared to electrolytic Nb foils.

The behavior observed here would thus suggest that surface critical currents present a marked anisotropy, with higher values for magnetic fields applied along the ripples in the irradiated samples. Further analysis of the surface superconducting characteristics and the effects of the different laser irradiation conditions can be better performed from *χ*_ac_(*H*) measurements because of their higher sensitivity, as discussed in the following section.

### 3.3. Surface Superconductivity Characterization

**Figure 7:**
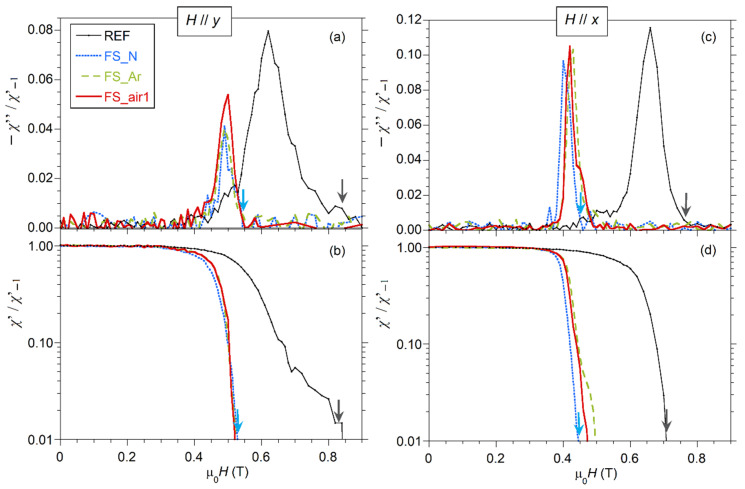
Components of the *χ*_ac_: *χ*″ (**a**,**c**) and *χ*′ (**b**,**d**), scaled by *χ*′_−1_, as a function of the DC magnetic field, μ_0_*H*, applied parallel to the *y*-axis (**a**,**b**) or the *x*-axis (**c**,**d**), at *T* = 5 K, *f* = 10 Hz and μ_0_*h*_0_ = 10 μT measured in descending fields from the initial field 1.5 T. The arrows mark μ_0_*H*_c3_ values for samples REF (black arrow) and FS_N (blue arrow), derived as explained in the text.

*H*_c3_ values can be estimated from *χ*_ac_(*H*) following a procedure similar to that described in [10,11,42]. The complex *χ*_ac_(*H*) curve was measured in descending DC magnetic fields applied parallel to the Nb sheet surface, starting from normal-state conditions and then approaching the superconducting transition by decreasing the DC magnetic field. Low frequency (*f* = 10 Hz) and low amplitude (μ_0_*h*_0_ = 10 μT) of the AC alternating magnetic field were used for the present measurements, similarly to those reported in references [10,11], for ease of comparison. Figure 7 shows *χ*′(*H*) and *χ*″(*H*) curves measured at 5 K with decreasing fields from 1.5 T down to 0 T for different samples and for two orientations of the external magnetic field. Note that the value of the initial DC magnetic field (μ_0_*H* = 1.5 T) was chosen considerably larger than μ_0_*H*_c2_ to ensure that the entire sample, including its surface, was in the normal state at the beginning of each measurement run [11]. All values in the graphs have been scaled by *χ*′_−1_, which is the value of *χ*′ measured at zero field, and it is close to the expected value for perfect diamagnetism for each sample, i.e., *χ*′_−1_(emu) ≈ *V*(cm^3^)/(4π), where *V* is the volume of the sample. *H*_c3_ at each temperature can be defined as the first deviation point from the values at normal state of *χ*″ or as the onset of *χ*′ (above noise level). In the figure, arrows indicate the position of the *H*_c3_ values of two of the samples (REF and FS_N), for clarity purposes.

From Figure 7, it is worth pointing out that fs-laser processing produces a pronounced shift of the transition towards smaller magnetic fields together with much narrower transitional widths, as compared to the reference sample. Two non-irradiated samples were measured, showing very similar curves (see Figure S1 presented in Supplementary Material), thus confirming this laser-modified behavior. The onset of the normal-to-superconducting transition of fs-laser processed samples occurs at higher fields when *H* is applied parallel to the nanostructures produced by the laser (*y*-axis) than when it is applied perpendicular to them (*x*-axis). The effect of the used atmosphere during laser processing is not significantly relevant, although some differences are observed, especially for fields applied along the *x*-axis (Figure 7c,d). The non-irradiated sample REF also shows some anisotropy, assigned to the elongated defects/grains produced by the rolling during its manufacture. Note that the maximum of *χ*″(*H*) curves is lower (thus indicating lower AC losses, which represent the energy dissipation during a cycle), for the fs-laser processed samples, especially for those irradiated in Ar and N_2_ atmosphere and for magnetic fields applied parallel to the induced nanostructures (*y*-axis). This could suggest lower surface resistance in these samples and conditions [43].

**Figure 8:**
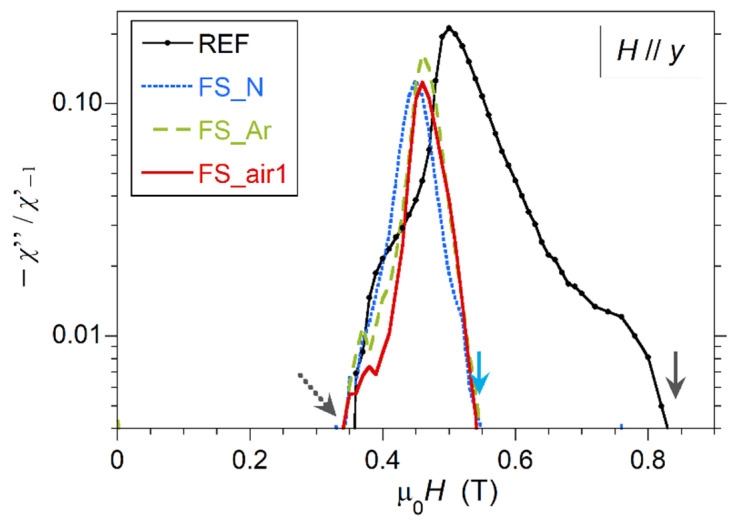
*χ*″(*H*) for the same samples and under the same conditions as in Figure 7a, but in this case μ_0_*h*_0_ = 100 μT. *χ*″ is plotted in a log scale. The dotted arrow indicates the estimated μ_0_*H*_c2_ value for these samples. Note that the two continuous arrows point to the same DC field values as in Figure 7a, to allow for a direct comparison between the two sets of data.

*χ*″(*H*) curves also allow the estimation of *H*_c2_, which is defined as the point where *χ*″(*H*) decreases to zero after the transition when ramping down the field [10,11]. As seen in Figure 7a, the increase of *χ*″(*H*) around *H*_c2_ is smooth at these conditions, so that we can only have an estimate of this value in the range between 0.35 T and 0.40 T in all samples, as the low-intensity signal makes the noise relevant, thus preventing more precise estimation of *H*_c2_ here. Nevertheless, it is possible to derive *H*_c2_ values using higher amplitude of the AC field, since the directly measured signal by the magnetometer is proportional to *h*_0_·*χ*_ac_(*H*, *f*, *h*_0_), so that the signal-to-noise ratio increases with *h*_0_. Figure 8 compares the same samples and conditions as in Figure 7a but for μ_0_*h*_0_ = 100 μT. *H*_c2_ values defined this way would be about 0.34 T for all the samples (dotted arrow in the figure). This is in good agreement with the result obtained previously from the heat capacity measurement shown in Figure 6b. It must be pointed out that, as expected, *H*_c2_ does not depend on the field orientation, since similar *H*_c2_ values are derived when the field is oriented parallel to the *x*-axis (see Figure S2 in the Supplementary Material).

The choice of small μ_0_*h*_0_ values in these measurements aims at improving the surface sensitivity. It can be observed that the onset of the normal-to-superconductor transition does not vary significantly by increasing μ_0_*h*_0_ from 10 to 100 μT, for laser processed samples, but it shifts at slightly lower external DC fields for the reference sample. This indicates that both AC field amplitudes would provide good sensitivity to analyze near-surface regions. It should be underlined that, upon increasing μ_0_*h*_0_, the AC field will sense deeper into the sample so that the peak of the *χ*″(*H*) curve shifts towards lower fields [5]. More specifically, for the fs-laser treated samples, the peak shifts from 0.49–0.50 T for the μ_0_*h*_0_ = 10 μT AC field down to the 0.45–0.46 T for the 100 μT AC field, when *H* is parallel to the *y*-axis (Figure 7 and Figure 8). The behavior is similar in all laser treated samples, although the peak of the sample processed in argon appears at slightly higher fields. In the case of the non-irradiated sample, the observed shift is larger, as the *χ*″(*H*) peak moves from 0.60 T (at 10 μT AC field) down to 0.50 T (at 100 μT AC field). In all samples, these fields are well above the μ_0_*H*_c2_ field, thus clearly indicative of surface phenomena.

**Figure 9:**
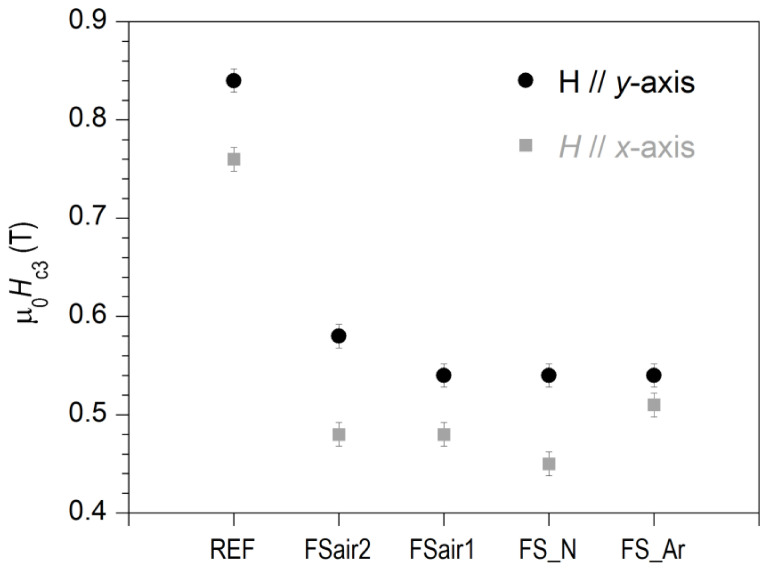
Critical surface fields, μ_0_*H*_c3_, of all analyzed samples derived from *χ*″(*H*) measurements at *T* = 5 K, *f* = 10 Hz and μ_0_*h*_0_ = 10 μT, with the external magnetic field applied parallel to the *x*- and *y*-axes.

The surface critical fields, *H*_c3_, derived from *χ*″(*H*) measurements at 5 K and AC fields of 10 Hz and amplitude 10 μT are plotted in Figure 9 for all analyzed samples presented in this study and for both DC field orientations. Note that the values *H*_c3_ obtained from *χ*′(*H*) curves, which are not displayed here for clarity purposes, show the same trends, but are slightly shifted towards lower magnetic fields than those corresponding to *χ*″(*H*). The estimated μ_0_*H*_c3_ values for external magnetic fields applied parallel to the surface nanostructures (*y*-axis) decrease from 0.84 T for the REF sample to about 0.54–0.58 T. That is, the *r*_32_ factor (=*H*_c3_/*H*_c2_) decreases from ≈2.4 for the as-rolled reference sample to 1.6–1.7 for fs-laser irradiated samples. Moreover, *H*_c3_ values are smaller when applying the magnetic field perpendicular to the nanostructures (*x*-axis). For this field orientation, we observe some changes depending on the processing atmosphere, with samples irradiated in argon and nitrogen having the smallest and the largest anisotropy, respectively. More precisely, for this field orientation, the transition of FS_N shifts slightly towards lower fields compared to the other irradiated samples (see Figure 7d), whereas the transitions of FS_air1 and FS_Ar are very similar for *χ*′/*χ*′_−1_ > 0.06, but differ on the onset of diamagnetism, which occurs at higher DC fields for FS_Ar, thus exhibiting a larger *H*_c3_ value. The reasons for the observed differences among the irradiated samples are still unknown. They are, in any case, small compared with those found between fs-laser irradiated and non-irradiated samples. It seems clear, however, that irradiation with fs-lasers, as used in this work, is sufficiently short in time to limit the chemical reaction between niobium and oxygen or nitrogen, thus resulting in very similar critical surface fields, independently of the atmosphere used during the irradiation process. Hence, this similarity points toward a topographic effect of the LIPSS here. Moreover, it was observed that the laser polarization orientation with respect to the rolling direction of the sample does not show a very important effect on the superconducting properties of the laser processed surface as the laser treatment diminishes rolling defects and both FS_air1 and FS_air2 samples exhibit a similar nanostructure and magnetic behavior.

Note that the effect of fs-laser processing on *H*_c3_ is similar to the effect of electropolishing and buffered chemical polishing of Nb wire reported by Sung et al. [11]. In that study, the authors observed a decrease of *H*_c3_ in all polished samples as compared with the as-drawn wires. For example, μ_0_*H*_c3_ at 5 K was reduced from ≈0.84 T (as-drawn) to ≈0.68 T after electropolishing for 3 h. They also found some differences in *H*_c3_ and *r*_32_ values by annealing (at 800 °C) or by baking (at 120 °C) the niobium samples. Following these results, it is thus worth further exploring the effect of different LIPSS topographies, together with annealing and/or baking of the Nb samples, on the surface superconducting properties of niobium.

## 4. Conclusions

Laser-induced periodic surface structures (LIPSS) were generated on oppositely aligned (3–4) mm × (3–4) mm areas on the top and bottom surfaces of as-rolled commercial niobium foils using two different fs-laser systems under different atmospheres (air, argon, and nitrogen). After laser treatment, the surface roughness is very similar in all cases. The formed low spatial period LIPSS (LSFL) have similar modulation depths (peak-to-valley distance of about 200 nm) and are aligned perpendicular to the laser beam polarization. They are characterized by a spatial frequency that depends on the laser wavelength: 775 nm (std 68 nm) and 570 nm (std 35 nm), i.e., approximately 73–74% of the irradiation wavelengths of λ = 1030 and 790 nm, respectively. High spatial frequency LIPSS (HSFL) with periods between 50 and 80 nm, are also formed between the LSFL structures, in the perpendicular direction.

Chemical analyses by EDX and XPS indicated some laser-induced oxidation effects, with Nb_2_O_5_ being the dominating type of oxide. Upon fs-laser processing in ambient air, a thin surface oxide layer of a few tens of nanometers extent was detected through cross-sectional STEM/EDX analyses. When laser processing takes place under inert gases, however, the laser-induced oxide layer thickness of ~5 nm is similar to that found for the native oxide on the non-irradiated reference sample. A small nitrogen signal is observed by EDX and XPS on the surface of the laser irradiated sample under nitrogen, not being consistent with the definite presence of niobium nitrides.

The surface critical field, *H*_c3_, was derived from AC susceptibility measurements as a function of the externally applied DC magnetic field, which was applied parallel to the surface and in both orientations with respect to the generated LIPSS. Clear effects of laser irradiation on the surface superconducting properties of the niobium foil samples have been shown. In particular, *H*_c3_ decreases for laser irradiated samples as compared to the non-irradiated reference sample. Laser irradiation also results in significantly narrower normal-superconductor transitions and lower *χ*″(*H*) peaks, whereas the upper critical field, *H*_c2_, remains unaffected. The microstructural anisotropy of the fs-laser-generated LIPSS is clearly reflected on the surface superconducting properties of the samples, with higher *H*_c3_ when the external magnetic field is parallel to the LIPSS (LSFL). Nevertheless, the differences in the spatial periodicity of LIPSS between these samples do not affect significantly the *H*_c3_ values. Moreover, it seems clear that irradiation with ultra-short pulsed lasers, as used in this work (with pulse durations of 280 fs and 30 fs), is of sufficiently short interaction time to limit the chemical reaction between niobium and oxygen or nitrogen, thus resulting in similar critical surface fields independently of the irradiation atmosphere.

The observed behavior indicates that fs-laser processing is useful to control the surface superconducting properties of niobium and could be an alternative to some well-stablished procedures, such as electropolishing or buffered chemical polishing. This work demonstrates that surface modification associated with ultra-short pulse laser processing strongly affects surface superconductivity. In order to understand completely the interaction between the laser generated surface nanostructures and superconductivity, additional studies using different laser sources (wavelengths or longer pulse durations) should be necessary.

## Figures and Tables

**Table 1:** Laser processing parameters used in 25 μm thick Nb foil samples irradiated with fs-lasers.

Sample	Laser	Atm.	*P* (W)	*f*_rep_ (kHz)	*v*_L_ (mm/s)	*d*_s_ (μm)	*F*_p_ (J/cm^2^)	*I*_p_ (GW/cm^2^)	*F*_2D_ (J/cm^2^)
FS_Ar	L1	Ar	0.18	1	1	1000	0.61	2671	17.1
FS_N	L1	N_2_	0.18	1	1	1000	0.61	2671	17.1
FS_air1	L2	Air	0.02	1	6	20	0.15	5023	16.6
FS_air2	L2	Air	0.02	1	6	20	0.15	5023	16.6

## Supplementary Materials

The following are available online at https://www.mdpi.com/2079-4991/10/12/2525/s1, Figure S1: Comparison between *χ*′(*H*) and *χ*″(*H*) curves of two different non-irradiated samples, REF and REF(2), nominally identical. Magnetic fields were applied parallel to the *y*-axis and the measurements were performed at 5 K, 10 Hz and μ_0_*h*_0_ = 10 μT with descending fields from the initial field of 1.5 T. Figure S2: *χ*″(*H*) for different samples at 5 K, 10 Hz and μ_0_*h*_0_ = 100 μT, measured in descending DC fields from the initial magnetic field 1.5 T. AC and DC magnetic fields were applied parallel to the *x*-axis.
