# Supplementary material for: Surface Superconductivity Changes of Niobium Sheets by Femtosecond Laser-Induced Periodic Nanostructures
Source: Nanomaterials (Basel). 2020 Dec 16;10(12):2525. doi: 10.3390/nano10122525 (PMC7765670; doi:10.3390/nano10122525)
Supplement: Supplementary file 1 [file nanomaterials-10-02525-s001.pdf]

nanomaterials

Supplementary

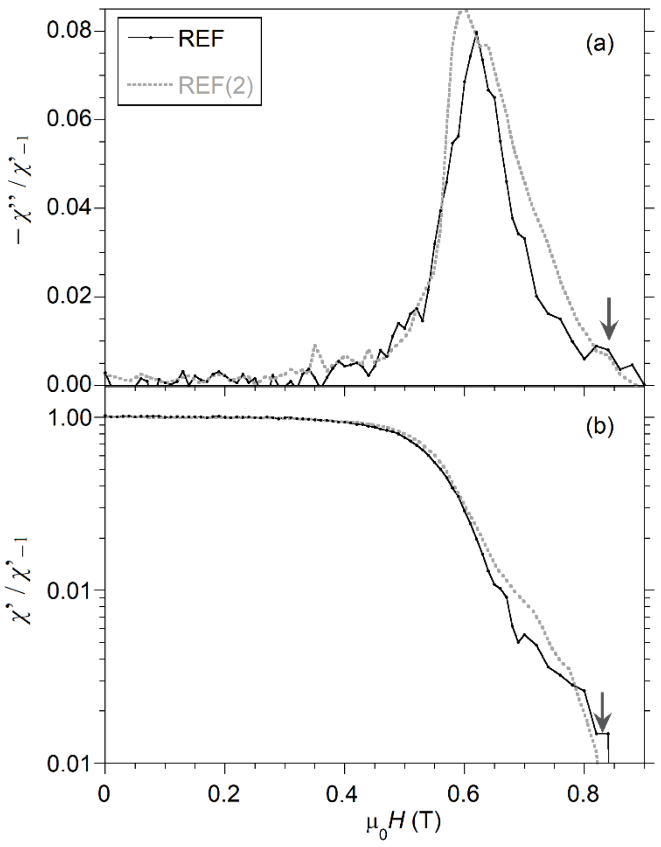

Figure S1. Comparison between $\chi'(H)$ and $\chi''(H)$ curves of two different non-irradiated samples, REF and REF(2), nominally identical. Magnetic fields were applied parallel to the *y*-axis and the measurements were performed at 5 K, 10 Hz and $\mu_0 h_0$ = 10 μT with descending fields from the initial field of 1.5 T. The arrows mark the $\mu_0 H_{c3}$ values.

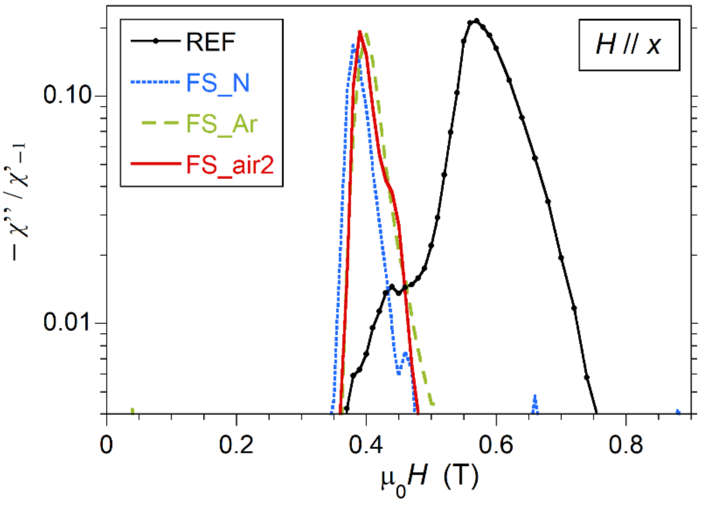

Figure S2. $\chi''(H)$ for different samples at 5 K, 10 Hz and $\mu_0 h_0$ = 100 μT, measured in descending DC fields from the initial magnetic field 1.5 T. AC and DC magnetic fields were applied parallel to the *x*-axis.
